# The Association between Air Pollution and Population Health Risk for Respiratory Infection: A Case Study of Shenzhen, China

**DOI:** 10.3390/ijerph14090950

**Published:** 2017-08-23

**Authors:** Xiaolin Xia, An Zhang, Shi Liang, Qingwen Qi, Lili Jiang, Yanjun Ye

**Affiliations:** 1 College of Geomatics, Shandong University of Science and Technology, Qingdao 266590, China; xiaxl@lreis.ac.cn (X.X.); qiqw@igsnrr.ac.cn (Q.Q.); 2 State Key Laboratory of Resources and Environmental Information System, Institute of Geographic Sciences and Natural Resources Research, Chinese Academy of Sciences, Beijing 100101, China; jiangll@igsnrr.ac.cn (L.J.); yeyj.14b@igsnrr.ac.cn (Y.Y.); 3 Shenzhen Prevention and Treatment Center for Occupational Disease, Shenzhen 518020, China

**Keywords:** air pollution, hospital admission, acute respiratory infection, time series study

## Abstract

Nowadays, most of the research on air pollution and its adverse effects on public health in China has focused on megacities and heavily-polluted regions. Fewer studies have focused on cities that are slightly polluted. Shenzhen used to have a favorable air environment, but its air quality has deteriorated gradually as a result of development in recent years. So far, no systematic investigations have been conducted on the adverse effects of air pollution on public health in Shenzhen. This research has applied a time series analysis model to study the possible association between different types of air pollution and respiratory hospital admission in Shenzhen in 2013. Respiratory hospital admission was divided into two categories for comparison analysis among various population groups: acute upper respiratory infection and acute lower respiratory infection. The results showed that short-term exposure to ambient air pollution was significantly associated with acute respiratory infection hospital admission in Shenzhen in 2013. Children under 14 years old were the main susceptible population of acute respiratory infection due to air pollution. PM_10_, PM_2.5_ and NO_2_ were the primary air pollutants threatening respiratory health in Shenzhen. Though air pollution level is generally relatively low in Shenzhen, it will benefit public health to control the pollution of particulate matter as well as other gaseous pollutants.

## 1. Introduction

Recent research studies have found that air pollution has significant direct and indirect adverse effects on public health in China [1,2,3]. The level of sensitivity to air pollution may vary among different population groups with different health conditions and ages [4,5,6,7]. Though such adverse effects on public health have proven to be aggravated as exposed concentration increases, there is little evidence to suggest a concentration threshold below which no adverse effects on public health can be expected [8,9]. The lowest concentration level at which those adverse effects start to manifest is not much greater than normal background concentration, which has been estimated to be 3–5 μg/m^3^ for PM_2.5_ in the United States and western Europe [9]. Nowadays, most of the research on air pollution and its adverse effects on public health in China have focused on megacities and heavily polluted regions including Beijing [10,11], Chongqing [12], Shanghai [13,14], Xi’an [15], and Wuhan [16,17], while few studies have focused on cities with relatively low pollution levels.

Shenzhen was once praised as an Environmental Protection Model City in 1997 for its favorable air environment. In recent years, as a member of the Pearl River Delta (PRD) region, which is one of the most developed regions with the highest aggregation of industry in China, Shenzhen has experienced deterioration in its air environment quality. Owing to the continuous development of industrialization and urbanization, severe air pollutions, including high concentrations of particulate matter (PM_10_ and PM_2.5_) [18,19,20,21], nitrogen dioxide (NO_2_) [21,22], and ozone (O_3_) [23,24] have been observed on both urban and regional scales in the PRD region. Simultaneously, the growing local industrial sector has fueled consumption, contributed to the rising number of vehicles (the vehicle amount had exceeded three million by the end of 2014 with the highest traffic density in China), and also has generated a certain amount of air pollutants in Shenzhen city [25]. Influenced by local pollution as well as the pollution from surrounding areas, the air quality in Shenzhen has deteriorated gradually and affected the living environment of local people to some extent. Zhang et al. [26] have evaluated the associations between PM pollution and all-cause mortality in Shenzhen in 2013. They have proved that effect of PM pollution on mortality was significant in Shenzhen, especially for elder and male groups. In this research, we intend to investigate possible associations between air pollution (SO_2_, NO_2_, PM_10_ and PM_2.5_) and hospital admissions for respiratory disease in Shenzhen in 2013. 

## 2. Data and Methods 

### 2.1. Air Pollutuion Data

**Figure 1:**
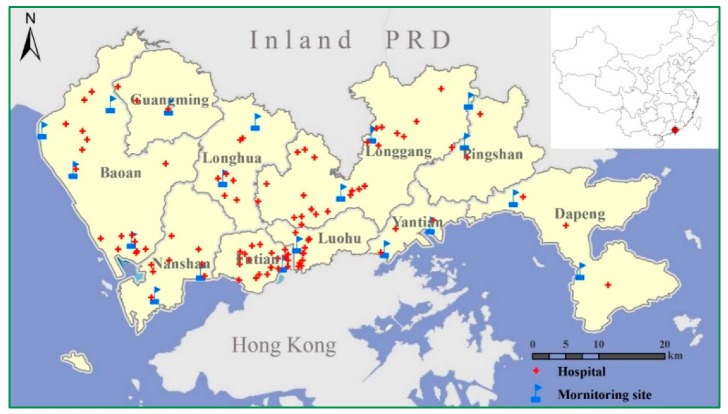
Locations of monitoring sites and hospitals in Shenzhen.

After the implementation of the National Ambient Air Quality Standard (GB3095-2012) [27] in China, the Shenzhen Environmental Monitoring Center constructed a systematically monitoring network that provides real-time monitoring hourly concentrations of several air pollutants (SO_2_, NO_2_, PM_10_ and PM_2.5_) to the general public since 1 January 2013. This monitoring network is composed of 19 monitoring stations scattered around the entire city (Figure 1). In this study, air pollution data was provided by our previous study on air pollutants in Shenzhen [28]. In the previous study, daily mass concentrations of air pollutants were collected at each site from 1 January to 31 December in 2013. Interpolations were carried out for each air pollutant on every single day. In this study, the daily concentration of each air pollutant was calculated as an average of the interpolated result of the whole study area. 

### 2.2. Hospital Admission Data

Hospital admission data was obtained from the Shenzhen Center for Medical Information. It contains 111,436 total respiratory hospital admission records (from 1 January 2013 to 31 December 2013) from 98 hospitals spread across the whole city area. These records contain the date of admission, age, gender, and discharge diagnosis from the tenth revision of the international classification of diseases (ICD-10, Ministry of Health Statistical Information Center, 2001) for each patient. Based on the ICD code, we picked out 35,277 acute respiratory hospitalized cases and divided them into acute upper respiratory infection (J00–J06) and acute lower respiratory infection (J20–J22) for analysis.

### 2.3. Statistical Analysis Model for Time Series Study

The core statistical model used in this research is the Generalized Additive Model (GAM), which is designed for exploring and fitting non-linear relationships between a response and predictor variables. In this research, we applied it to explore the relationship between air pollution and corresponding effects on respiratory health. While the effect of the air pollution on human health is not limited to the exposure period, it usually shows up in a lag of time. Therefore, we combined the GAM with a lag model—the Distributed Lag Model (DLM)—to examine the lag effect of air pollutants. The DLM was proposed to evaluate the lagged effect when the outcome in a specific time is influenced by the level of the predictor in previous times, up to a maximum lag, and it has recently been used to quantify health effects study associated with air pollution in the field of epidemiology [29,30,31,32]. The main advantage of the DLM is that it allows the model to contain a detailed representation of the time-course of the exposure–response relationship, which in turn provides an estimate of the overall effect as the sum of the single lag effects upon the whole lag period considered. Besides the air pollutants, we also added several confounding variables into consideration [33,34,35]. We used smoothing spline functions of calendar time and temperature, pressure, as well as relative humidity, to adjust for long-term trends and control for the potential confounding effects of weather, respectively. Degrees of freedom (df) of smoothing functions were determined by the Akaike’s information criterion. We also applied a generalized cross validation (GCV) to guide the determination of df until the absolute values of the sum of errors achieved a minimum. We also included a dummy variable for day of the week. The model was of the form:

$$\log\left[E\left(Y_{t}\right)\right]=\alpha +\mathrm{DOW}+\overset{L}{\underset{i=1}{\sum }}\beta_{i}X_{t-L,i}+S\left(\mathrm{time},\mathrm{df}\right)+S\left(Z_{t},\;\mathrm{df}\right)$$

where *t* is the day for observation; $E\left(Y_{t}\right)$ is the expected number of daily respiratory hospital admission on day *t*; α is the intercept term; DOW is the indicator variable for the day of week, as the dummy variable; β represents the log-relative rate of hospital admission associated with a unit increase of air pollutants; $X_{t-L}$ indicates the pollutant concentrations at *L* days before day *t*; S(time,df) represents the smoothing function of calendar time (df = 7); and $S\left(Z_{t},\;\mathrm{df}\right)$ represents the smoothing functions of the meteorological variables, in this case average temperature (df = 3), pressure (df = 5), relative humidity(df = 3), respectively.

We carried out a time series analysis in terms of acute upper respiratory infection and acute lower respiratory infection. The accumulative lag effects were examined with a lag of 14 days for each air pollutant. We also evaluated the lag effects on different population groups. The Distributed Lag Non-linear Model (DLNM) and Mixed GAM Computation Vehicle (MGCV) packages in R (3.4.0, University of Auckland, Auckland, New Zealand) were applied to construct the analytic model. All results were presented as relative risk (RR) or percent change in daily hospital admission amount and its 95% confidence interval (CI) in association with a 10 μg/m^3^ increase of air pollutant concentrations.

## 3. Results and Discussion

### 3.1. Statistical and General Analysis

Table 1 and Table 2 summarize the statistical characteristics of hospital admissions, air pollutants, and meteorological factors in Shenzhen in 2013. There were a total of 35,277 respiratory hospital admissions at the 98 hospitals, of which 11,994 cases were due to upper respiratory infection and 23,283 cases were due to lower respiratory infection; the number of male hospitalizations was twice as great as that of female hospitalizations, and admissions for patients younger than 14 years comprised 80% of the total. On average, there were approximately 100 acute respiratory hospital admissions per day in the study area, among which 34 cases were due to acute upper respiratory infection, and 66 cases were due to acute lower respiratory infection. During the study period, the annual average concentrations of SO_2_ and NO_2_ were 13 and 39 μg/m^3^, respectively, which are both below the Chinese ambient air quality standards (60 μg/m^3^ for SO_2_ and 40 μg/m^3^ for NO_2_). The annual average concentrations of PM_10_ and PM_2.5_ were 62 and 43 μg/m^3^, respectively, and PM_2.5_ exceeded the Chinese ambient air quality standards (70 μg/m^3^ for PM_10_ and 35 μg/m^3^ for PM_2.5_), making particulate matter the main air contaminant in Shenzhen in 2013. Nevertheless, the pollution level of particulate matters in Shenzhen was still lower than most of the megacities in China [36,37,38]. For meteorological conditions during the study period, daily average temperature and humidity were 23.5 °C and 75.5%, respectively, reflecting the subtropical oceanic climate of Shenzhen.

**Figure 2:**
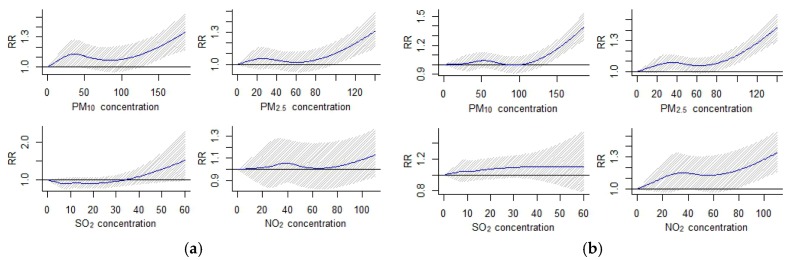
The smoothed exposure response curves of air pollutant concentrations in a lag of 10 days against the relative risk (RR) of acute respiratory infection hospital admission. (**a**) Acute upper respiratory infection; (**b**) Acute lower respiratory infection.

Firstly, a smoothing function in the GAM was used to graphically analyze the exposure–response relationship between air pollutant concentrations and hospital admission of two kinds of acute respiratory infections. Results (one for a lag of 10 days is shown in Figure 2) showed that each air pollutant revealed a threshold value (Table 3) beyond which their effects on respiratory disease could be considered linear. Therefore, a threshold method was applied to evaluate the linear effects of air pollution in relation to 10-unit increases in air pollutant concentration beyond the threshold. In the meantime, experiments on different population groups all suggested the same threshold values for each air pollutant in both upper respiratory infection and lower respiratory infection. As a result, we adopted a uniform value for the threshold for each air pollutant in the following analysis.

**Figure 3:**
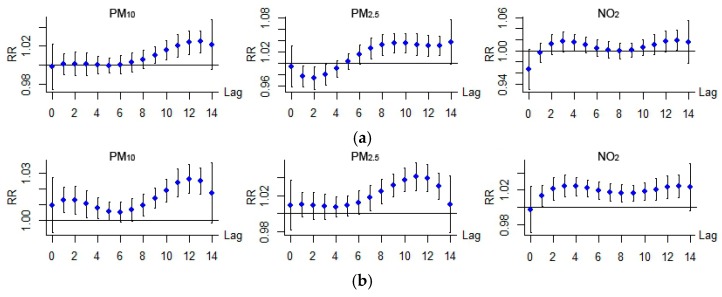
Lag–response relationship between air pollutant and RR for hospital admission of acute respiratory infection. (**a**) Acute upper respiratory infection; (**b**) Acute lower respiratory infection.

Table 3 shows the overall cumulative percentage increase of total hospitalizations due to acute respiratory infection associated with a 10-unit increase in air pollutant above the threshold over 14 days of lag, together with its 95% confidence interval for each air pollutant. Three air pollutants had significant lag effects; of these, PM_10_ generally had a lag of 8–13 days, PM_2.5_ had a lag of 7–13 days, NO_2_ had a lag of 1–13 days (Figure 3). Acute upper respiratory infection hospitalizations were significantly increased by 13.5% (95% CI: 5.6, 22) and 20.6% (95% CI: 5.6, 37.7) per 10 μg/m^3^ increases beyond the threshold in PM_10_ and PM_2.5_ respectively. Acute lower respiratory infection hospitalizations significantly increased by 22.8% (95% CI: 16.5, 29.3), 34.1% (95% CI: 21, 48.6) and 32.1% (95% CI: 20.5, 44.9) per 10 μg/m^3^ increases beyond the threshold in PM_10_, PM_2.5_ and NO_2_, respectively. No significant association was detected between SO_2_ and either acute respiratory infection, therefore, the lag–response plots were only exhibited for the other three pollutants. 

Since the contemporary effects of multiple pollutants may confuse the effect estimated for any single air pollutant, a multiple-pollutant model was applied to investigate the contemporary effect of different combinations of air pollutants. In order to guarantee the independence of each variable in the regression model, only irrelevant air pollutants (R^2^ < 0.7 in Table 4) were selected for analysis. Table 5 compares the results of the single-pollutant and multiple-pollutant models. For SO_2_, its effects maintained insignificance after adding the other air pollutants for adjustment; for PM_10_, PM_2.5_ and NO_2_, their effects became insignificant when adding SO_2_ for adjustment; for the rest of the combinations, there were no significant changes of effects after adding other pollutants for adjustment. All in all, no additive effects were detected among any pollutant combinations. 

### 3.2. Comparison among Different Groups

**Figure 4:**
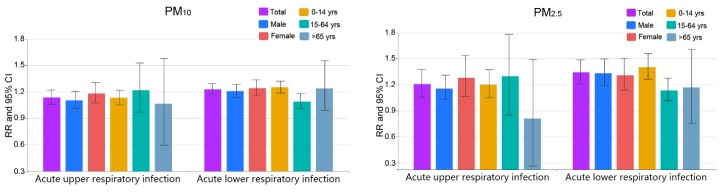

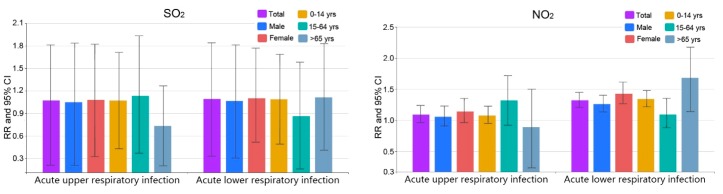
Relative Risks (RRs) and 95% confidence intervals (CIs) of acute respiratory infection hospital admissions in associations with 10 μg/m^3^ increases in air pollutant concentrations in Shenzhen, 2013.

In this section, we explored the effects of air pollution on acute respiratory infection in terms of gender and age groups (Figure 4). Regarding gender, significant associations were detected between acute upper respiratory infection and PM_10_ and PM_2.5_; and acute lower respiratory infection and PM_10_, PM_2.5_ and NO_2_ across both male and female groups. The RR estimated for both kinds of hospitalizations tended to be smaller for males than for females, except for the acute lower respiratory infection hospitalizations associated with PM_2.5_. While their confidence intervals were overlapping, gender difference was not statistically significant in this situation. Regarding age, for acute upper respiratory infection, significant associations were only detected for patients under 14 years with PM_10_ and PM_2.5_. For acute lower respiratory infections, significant associations were detected for patients under 14 years and patients aged from 15 to 64 years with both PM_10_ and PM_2.5_, and for patients under 14 years and patients above 65 years with NO_2_. The RR estimated for acute lower respiratory infection hospitalizations tended to be smaller for patients aged above 14 years than those aged under 14 years associated with both PM_10_ and PM_2.5_, and smaller for patients aged under 14 years than those aged above 65 years associated with NO_2_.

**Figure 5:**
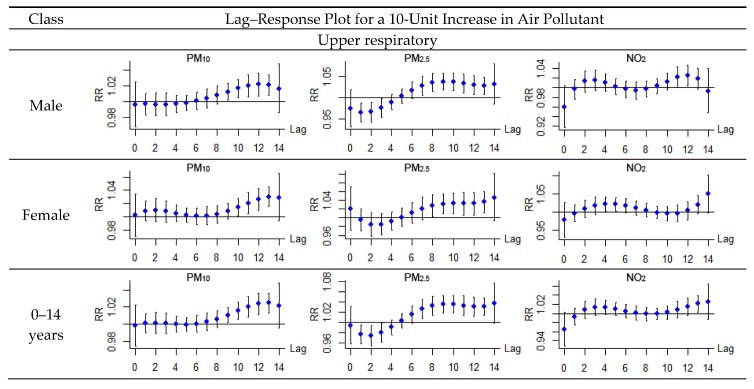

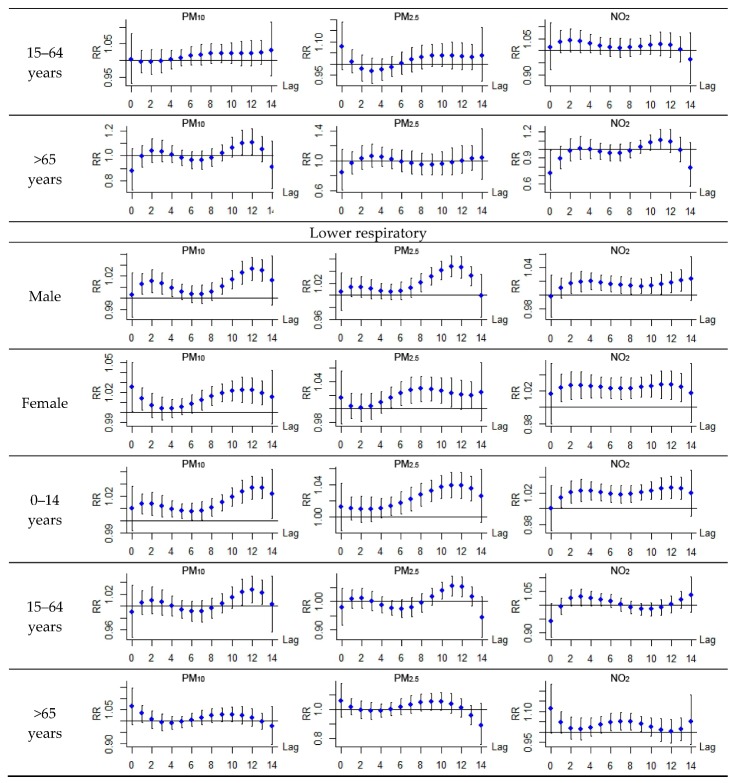
Lag–response relationship between air pollutants and the RR for acute respiratory infection in different population groups.

Figure 5 shows the lag-response plots for different population groups. For acute upper respiratory infection, regarding gender, no obvious distinctions were observed between the male group and the female group. Significant associations both emerged in a lag of 9–10 days and 7–8 days, disappeared in a lag of 13 days for PM_10_ and PM_2.5_ respectively, and no associations for NO_2_. Regarding age, significant associations were only detected for patients under 14 with PM_10_ (in a lag of 9–13 days) and PM_2.5_ (in a lag of 7–13 days), no associations for patients in the other two age groups were detected with any air pollutant. Acute lower respiratory infection revealed slightly different patterns for the male group and the female group: significant associations were detected in a lag of 1–4 days and 9–13 days for the male group, while the lag was 0–1 days and 7–13 days for the female group with PM_10_, 8–13 days for the male group and 5–11 for the female group with PM_2.5_, and 2–13 days for the male group and 1–13 for the female group with NO_2_. The lag time for the female group was shorter than the male group when considering acute lower respiratory infection. Regarding age, for patients under 14 years, significant associations were detected with PM_10_ and PM_2.5_ and NO_2_ with lags of 1–14 days, 5–13 days and 1–13 days, respectively; for patients aged from 15 to 64 years, significant associations were detected with PM_10_ and PM_2.5_ with lags of 11–13 days and 10–12 days, respectively; for patients older than 65 years, a significant association was detected with NO_2_ and a lag of 6–9 days. The specific percentage increase of hospital admissions over a lag of 14 days in association with 10 μg/m^3^ increases in air pollutant concentrations beyond the threshold for different population groups is shown in Table 6.

### 3.3. Discussion

This study intended to explore the associations between the effects of single air pollutant and acute respiratory infection hospital admission. We carried out a series of experiments in terms of acute upper respiratory infection and acute lower respiratory infection among different population groups. For acute upper respiratory infection, significant associations were only observed for patients under 14 years with PM_10_ and PM_2.5_. For acute lower respiratory infection, significant associations were observed for patients under 14 years and patients aged from 15 to 64 years with both PM_10_ and PM_2.5_, and for patients under 14 years and patients above 65 years with NO_2_. The estimated relative risk (RR) values tended to be higher for patients aged under 14 years or above 65 years than for those aged from 15 to 65 years.

The exposure–response relationship is crucial for public health assessment, and there has been increasing demand for presenting the relevant curves. The relationships may vary by study areas, depending on factors such as air pollution components, climate, and the health of the studied population [39]. In this study of Shenzhen, a non-linear exposure–response curve capturing the relationship between air pollutant concentration and the number of acute respiratory infection hospitalization was presented. It showed that air pollutant revealed significant adverse effects on respiratory health when exceeding a threshold concentration. For PM_10_, SO_2_ and NO_2_, the threshold values were even lower than the daily air quality standard in China (Table 7). Therefore, current air quality standard for the three pollutants might not be sufficient to protect the public health in Shenzhen. Further control of air pollution is likely to result in health benefits.

In other relevant studies in China, the total number of hospital admissions for respiratory diseases increased by 0.4–1.6%, 1.3–3.0%, and 1.8–3.0% for 10 μg/m^3^ increases in PM_10_, SO_2_ and NO_2_, respectively [40,41,42]. In Europe and the USA, a 10 μg/m^3^ increase in PM_10_, SO_2_ and NO_2_ resulted in 1.0–2.4%, 0.6–1.6% and 0.9–1.1% increases in the total number of hospital admissions for respiratory diseases [43,44,45]. All of these results present much weaker associations between air pollution and respiratory disease than what we have found in this study. The difference for the effect estimates between this study and other studies can be explicated in two aspects. On one hand, this study only focused on the high-pollution days by applying a threshold model; therefore, low-pollutant effects that may weaken the associations between air pollution and respiratory disease were ignored. On the other hand, the causes of respiratory diseases are various, some of which are completely unrelated to the atmospheric environment. Meanwhile, some diseases usually occur as a result of the long-term effect of air pollution. Therefore, using total respiratory disease for a short-term effect analysis may result in relatively weak association, and picking out specific respiratory disease categories in future work will be beneficial. Another study in Lanzhou city in China had explored associations between air pollutants and respiratory hospital admissions using specific respiratory disease categories, including upper respiratory infection, chronic obstructive pulmonary disease (COPD), and pneumonia. Their results proved that the association between air pollution and specific respiratory diseases was significantly stronger than that between air pollution and total respiratory diseases [33]. In the meantime, applying age stratification to explore effects of air pollution on different population groups also can improve the degree of accuracy.

Although the strongest evidence connecting air pollutants with adverse health effects at present is for PM, this study found similarly strong health effects for NO_2_, which suggest that it is also an important factor influencing public health in the polluted air mixture in Shenzhen. Although it is proved that NO_2_ may contribute to PM formation, several studies suggest that they are also separately regulated pollutants independently related with adverse health effects in China. Chen et al. reported that out of all of the pollutants they examined, only NO_2_ remained significantly associated with daily mortality after adjustment for any co-pollutants in Shanghai [13]. 

There are still several limitations in our study. Although hospital admissions in China are usually unscheduled, we were not able to exclude the scheduled ones. As in most relevant studies, we simply averaged the pollution levels of the whole city as the proxy for population exposure level to air pollution. The simple averaging method may raise a number of issues given that pollutant concentrations can differ from location to location, and that ambient pollutant concentrations differ from personal exposure level to air pollution level [46]. The differences between these proxy values and the true exposures generate an inherent and unavoidable type of measurement error in time-series air pollution studies. The use of personal exposure monitors may help to address this issue; however, the cost of using personal exposure monitors is too high under the current circumstances in China. Meanwhile, compared with other air pollution studies in Europe and North America, the data we collected was limited in that it was from only one city and over one year, which may lead to unexpected errors due to its special geographical location or exceptional events, such as flu outbreak. We also failed to control for seasonality. To improve the reliability and accuracy of the analysis, long-term data should be collected for at least two years in more cities, and seasonality control should be added in the model for further study. 

## 4. Conclusions

In summary, short-term exposure to ambient air pollution was associated with acute respiratory infection hospital admission in Shenzhen in 2013. Besides the well-known adverse effects of particulate matter pollution, NO_2_ also had considerable health effects in Shenzhen, and it also increased the number of hospitalizations due to acute respiratory infection in Shenzhen. Children under 14 years old were the main susceptible population of acute respiratory infection due to air pollution. Stronger effects were observed for females than for males. PM_10_, PM_2.5_ as well as NO_2_ were the primary air pollutants threatening respiratory health in Shenzhen. Though air pollution level is generally relatively low in Shenzhen, it will benefit public health to control the pollution of PM as well as NO_2_.

## Figures and Tables

**Table 1:** Descriptive statistics of daily respiratory hospitalizations for Shenzhen (2013).

Item	Amount	Mean ± SD	Min	P (25)	Median	P (75)	Max
Hospital admission							
Acute upper respiratory	11,994	34 ± 9	12	28	33	40	63
Male	7193	20 ± 6	6	16	20	24	39
Female	4801	14 ± 5	2	10	13	16	30
0–14 years	10,633	30 ± 8	8	25	29	35	55
15–64 years	1204	3 ± 2	0	2	3	4	15
>65 years	157	0 ± 1	0	0	0	1	4
Acute lower respiratory	23,283	66 ± 12	29	57	66	74	102
Male	14,447	41 ± 8	16	35	41	46	66
Female	8836	25 ± 6	9	21	25	29	49
0–14 years	19,391	55 ± 11	27	46	55	62	88
15–64 years	2995	8 ± 4	0	6	8	11	21
>65 years	897	3 ± 2	0	1	2	3	10

P (25): Percentile of 25; P (75): Percentile of 75.

**Table 2:** Descriptive statistics for air pollutants and meteorological factors for Shenzhen (2013).

Item	Mean ± SD	Min	P (25)	Median	P (75)	Max
Air pollutant						
SO_2_ (μg/m^3^)	13 ± 5.5	5.0	8.1	10.5	14.2	53.4
NO_2_ (μg/m^3^)	39 ± 16.4	14.8	29.8	37.6	49.0	104.8
PM_10_ (μg/m^3^)	62 ± 34.8	10.3	35.4	50.3	80.7	184.8
PM_2.5_ (μg/m^3^)	43 ± 24.6	8.3	21.3	34.9	54.7	135.8
Meteorological factors						
Temperature (°C)	23.5 ± 4.9	9.8	19.8	24.6	27.7	31.2
Pressure (hPa)	1004.9 ± 6.1	986.8	1000.2	1004.6	1010.1	1019.2
Relative humidity (%)	75.5 ± 14.8	24	68	78	87	100

**Table 3:** Percentage increase of total hospital admissions associated with 10 μg/m^3^ increase in air pollutant concentrations within 14 days of lag for acute respiratory infections in Shenzhen in 2013.

Pollutant	Threshold	Upper Respiratory	Lower Respiratory
PM_10_	100 (μg/m^3^)	13.5 (5.6, 22.0) *	22.8 (16.5, 29.3) *
PM_2.5_	80 (μg/m^3^)	20.6 (5.6, 37.7) *	34.1 (21.0, 48.6) *
SO_2_	30 (μg/m^3^)	7.4 (−79, 81.3)	9.2 (−66.9, 84.1)
NO_2_	60 (μg/m^3^)	9.2 (−3.8, 24)	32.1 (20.5, 44.9) *

* *p* < 0.05.

**Table 4:** Correlation coefficients between daily air pollutant concentrations in Shenzhen in 2013.

R^2^	NO_2_	PM_10_	PM_2.5_
SO_2_	0.46	0.68	0.66
NO_2_		0.57	0.45
PM_10_			0.92

**Table 5:** Percent increase of total hospital admissions associated with 10 μg/m^3^ increase in air pollutants concentrations with single and multiple-pollutant models for acute respiratory infection in Shenzhen in 2013.

Pollutant	Adjustment	Upper Respiratory	Lower Respiratory
SO_2_	none	7.4 (−79, 81.3)	9.2 (−66.9, 84.1)
	NO_2_	7.2 (−67, 81.4)	7.9 (−51.3, 67.1)
	PM_10_	8.6 (−53.3, 70.5)	10.3 (−48.1, 68.7)
	PM_2.5_	6.9 (−60.5, 74.3)	8.7 (−41.1, 58.5)
NO_2_	none	9.2 (−3.8, 24)	32.1 (20.5, 44.9) *
	SO_2_	7.3 (−2.2, 26.7)	10.5 (−3.3, 23.2)
	PM_10_	8.6 (−1.8, 18.9)	25.1 (18.8, 31.4) *
	PM_2.5_	8.9 (−1.5, 19.2)	27.3 (19.2, 35.4) *
PM_10_	none	13.5 (5.6, 22.0) *	22.8 (16.5, 29.3) *
	SO_2_	7.3 (−2.2, 26.7)	10.5 (−3.3, 23.2)
	NO_2_	10.3 (1.2, 19.7) *	15.3 (8.2, 22.9) *
PM_2.5_	none	20.6 (5.6, 37.7) *	34.1 (21.0, 48.6) *
	SO_2_	7.3 (−2.2, 26.7)	10.5 (−3.3, 23.2)
	NO_2_	10.3 (1.2, 19.7) *	15.3 (8.2, 22.9) *

* *p* < 0.05.

**Table 6:** Percentage increase of hospital admissions in associations with 10 μg/m^3^ increase in air pollutant concentrations for different population groups in Shenzhen in 2013.

Infection	Group	PM_10_	PM_2.5_	SO_2_	NO_2_
Upper respiratory	Male	10.3 (1.2, 20.3) *	15.6 (1.3, 30.1) *	5.2 (−79.5, 83.6)	5.7 (−9.2, 23.0)
	Female	18.3 (7.3, 30.5) *	27.9 (6.5, 53.6) *	8.1 (−67.5, 82.2)	14.1 (−3.7, 35.1)
	0–14 years	13.1 (5.2, 21.6) *	20.2 (5.3, 37.3) *	7.1 (−57.0, 71.3)	8.0 (−5.0, 22.8)
	15–64 years	21.5 (−3.4, 52.7)	29.6 (−15.2, 78.1)	13.1 (−62.9, 93.3)	31.9 (−7.9, 71.9)
	>65 years	6.5 (−40.8, 55.9)	−19.1 (−73.8, 49.1)	−26.4 (−79.7, 26.7)	−10.7 (−76.5, 49.9)
Lower respiratory	Male	20.8 (13.7, 28.2) *	33.2 (18.7, 49.7) *	6.7 (−69.5, 81.3)	26.1 (13.4, 40.3) *
	Female	24.2 (15.6, 33.4) *	31.0 (13.9, 50.5) *	10.3 (−48.3, 77.0)	42.9 (26.6, 61.3) *
	0–14 years	25.1 (18.5, 32.0) *	40.2 (26.0, 56.1) *	9.1 (−50.8, 68.6)	34.2 (7.3, 30.5) *
	15–64 years	8.6 (1.5, 17.6) *	13.4 (0.6, 26.2) *	−13.7 (−83.9, 58.3)	9.2 (−11.8, 35.2)
	>65 years	23.7 (−1.4, 55.4)	16.9 (−24.5, 60.9)	11.4 (−58.9, 83.0)	68.1 (14.1, 147.6) *

* *p* < 0.05.

**Table 7:** Threshold concentration in Shenzhen and daily limit concentration in air quality standard in China.

Pollutant	Threshold	Days over Threshold	Daily Limit	Days over Limit
PM_10_	100 (μg/m^3^)	63	150 (μg/m^3^)	13
PM_2.5_	80 (μg/m^3^)	31	75 (μg/m^3^)	44
SO_2_	30 (μg/m^3^)	5	150 (μg/m^3^)	0
NO_2_	60 (μg/m^3^)	48	80 (μg/m^3^)	6
